# Low temperature exposure induces browning of bone marrow stem cell derived adipocytes *in vitro*

**DOI:** 10.1038/s41598-018-23267-9

**Published:** 2018-03-21

**Authors:** Ksenija Velickovic, Hilda Anaid Lugo Leija, Ian Bloor, James Law, Harold Sacks, Michael Symonds, Virginie Sottile

**Affiliations:** 1 Wolfson Centre for Stem Cells, Tissue Engineering and Modelling (STEM), School of Medicine, University Park - CBS Building, University of Nottingham, Nottingham, NG7 2RD United Kingdom; 2 The Early Life Research Unit, Division of Child Health, Obstetrics and Gynaecology, University Hospital, University of Nottingham, Nottingham, NG7 2UH United Kingdom; 3 Nottingham Digestive Disease Centre and Biomedical Research Centre, School of Medicine, University Hospital, University of Nottingham, Nottingham, NG7 2UH United Kingdom; 4 VA Endocrinology and Diabetes Division, VA Greater Los Angeles Healthcare System, and Department of Medicine, David Geffen School of Medicine, University of California, Los Angeles, CA 90073 USA

## Abstract

Brown and beige adipocytes are characterised as expressing the unique mitochondrial uncoupling protein (UCP)1 for which the primary stimulus *in vivo* is cold exposure. The extent to which cold-induced UCP1 activation can also be achieved *in vitro*, and therefore perform a comparable cellular function, is unknown. We report an *in vitro* model to induce adipocyte browning using bone marrow (BM) derived mesenchymal stem cells (MSC), which relies on differentiation at 32 °C instead of 37 °C. The low temperature promoted browning in adipogenic cultures, with increased adipocyte differentiation and upregulation of adipogenic and thermogenic factors, especially UCP1. Cells exhibited enhanced uncoupled respiration and metabolic adaptation. Cold-exposed differentiated cells showed a marked translocation of leptin to adipocyte nuclei, suggesting a previously unknown role for leptin in the browning process. These results indicate that BM-MSC can be driven to forming beige-like adipocytes *in vitro* by exposure to a reduced temperature. This *in vitro* model will provide a powerful tool to elucidate the precise role of leptin and related hormones in hitherto functions in the browning process.

## Introduction

Traditionally, two types of adipose tissue are recognized in mammals: white adipose tissue (WAT) and brown adipose tissue (BAT)^1^. Both types are specialized to store energy in the form of lipids, whilst BAT has capacity to dissipate energy in the form of heat, thereby contributing to thermogenesis in mammals^2,3^. Heat production in BAT is mediated by a unique uncoupling protein 1 (UCP)1 which stimulates proton conductance across the mitochondrial membrane to uncouple respiration from adenosine triphosphate (ATP) synthesis^1^. The most potent physiological stimulus to activate UCP1 is cold exposure, which has been shown to promote the appearance of UCP1 both *in vivo* and *in vitro*^4,5^. It is unclear, however, whether this thermal response could have a comparable impact on the regulation of stem cell differentiation into adipocytes. The importance of thermal stimulation in determining the composition of fat is emphasized from the fact that brown-like adipocytes can also appear at anatomical sites corresponding to WAT^6,7^ to form recruitable ‘beige’ adipocytes^8,9^. Unlike classic BAT, beige cells express much lower amounts of UCP1^10^, but after cold stimulation or treatment with ß-adrenergic receptor agonists, they resemble classic BAT and UCP1 is upregulated^10,11^. Moreover, when fully stimulated, beige adipocytes undergo UCP1-mediated respiration^10^, but their molecular and developmental characteristics are different from brown adipocytes. For example, in addition to *UCP1*, beige cells express a subset of specific markers such as *CD137*, *TMEM26*, *CITED1* and *P2RX5*, which is also recognised as an adipogenic regulator^10,12–17^.

Critically, all adipocytes are mesenchymal in origin^18,19^. Mesenchymal stem cells (MSCs) are present in a wide range of tissues, and used as a cellular model to study differentiation towards a variety of lineages^20^. Bone Marrow (BM) derived MSCs are characterised by their potential to differentiate into several cell types, including osteoblasts, chondroblasts, adipocytes and myocytes^21^. Recently, more complex roles have been attributed to BM adipocytes, not only as a passive fat depot, but also as cells actively participating in BM lipid metabolism and osteogenesis^22^, with features described as a mix of both WAT and BAT characteristics^23^. However, detailed characterization of BM-derived mMSCs and the mechanism enabling them to develop into brown, beige or white adipocytes have not previously been examined. MSCs have recently been proposed to provide a source of thermogenic cells that could ultimately be used to treat obesity and/or diabetes^24^. There is evidence that isolated cells can respond to changes in temperature directly^4^, suggesting that additional pathways, besides the sympathetic nervous system, may mediate brown adipogenesis by cold stimuli. An important step towards this goal is to establish the optimal conditions for inducing brown and/or white-to-brown adipogenesis from MSCs and to test our hypothesis regarding the effects of different temperature conditions on adipogenic differentiation *in vitro*. It is known that BM adipocytes secrete and express high levels of leptin and raised leptin expression is one marker of adipocyte differentiation^23^. Accordingly, we evaluated the potential of BM-derived mouse MSCs to undergo browning and the extent to which this can be modulated by the temperature at which the cells are incubated during differentiation, together with its potential impact on brown/beige markers and leptin expression.

## Results

### Mouse Mesenchymal stem cells (mMSCs) achieve an adipogenic phenotype and express both UCP1 and leptin

**Figure 1:**
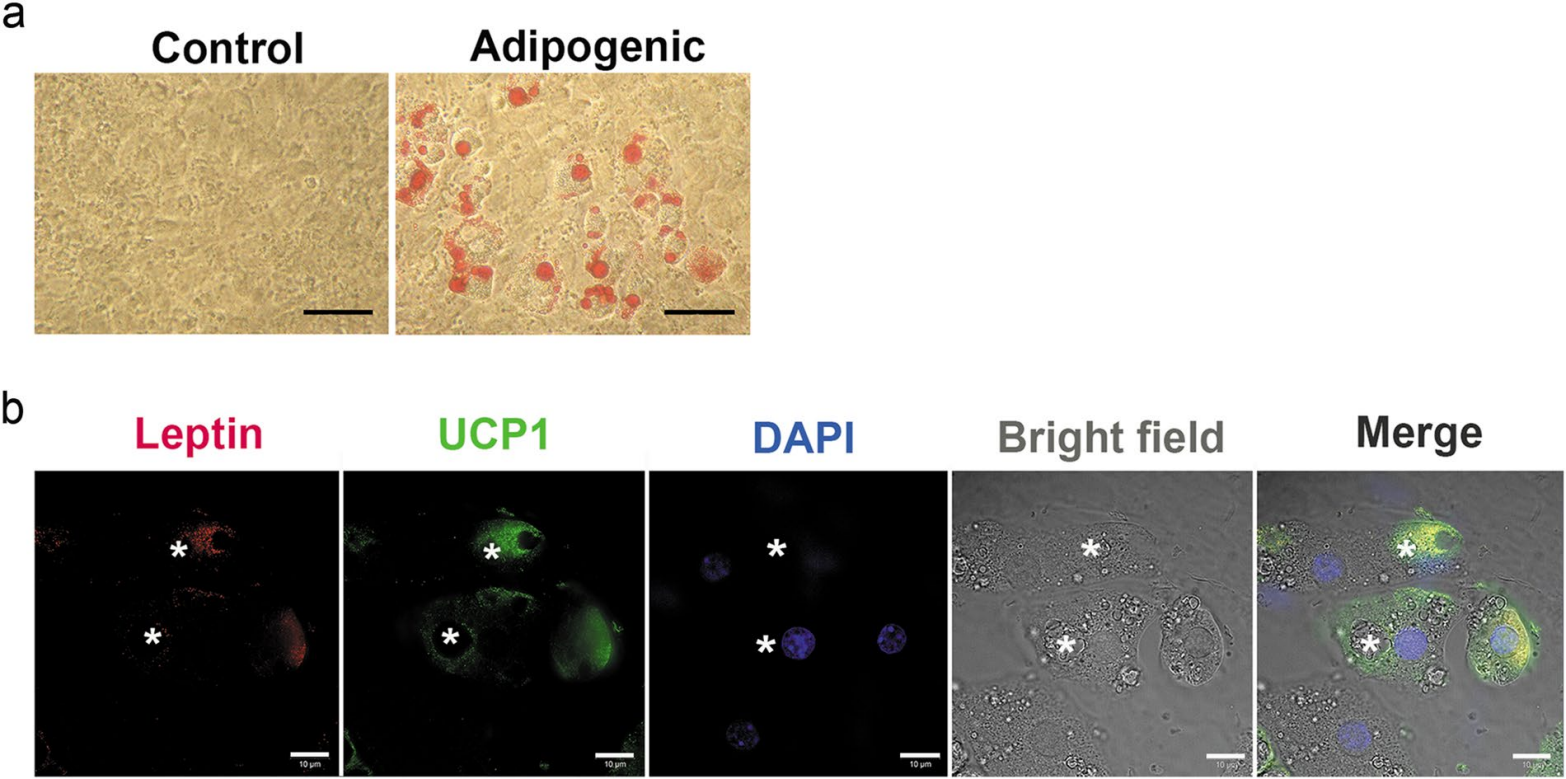
mMSCs differentiated adipocytes show uncoupling protein (UCP)1 and leptin expression. (**a**) mMSCs acquired an adipogenic phenotype confirmed by ORO staining. Scale bars: 50 μm. (**b**) Super-resolved structured illumination microscope image showing presence of leptin^+^/UCP1^+^ cells in AD-treated mMSC cultures. Leptin (red) and *UCP1* (green) were found to be co-expressed in differentiated cells (asterisks - LDs), with DAPI nuclear counterstain (blue). Scale bars: 10 μm. n = 3 individual experiments.

In order to examine the time course of mMSC differentiation, cells were treated with adipogenic medium at 37 °C. By day 9, cells were clearly distinguishable from untreated cells maintained in standard medium, as confirmed by oil red O (ORO) staining (Fig. 1a). To further characterize the adipogenic response, UCP1 and leptin immunostaining was performed. Both markers were observed to be expressed in the majority of differentiated cells (Fig. 1b).

### Enhanced adipogenesis and morphological changes show signs of browning in hypothermic conditions

**Figure 2:**
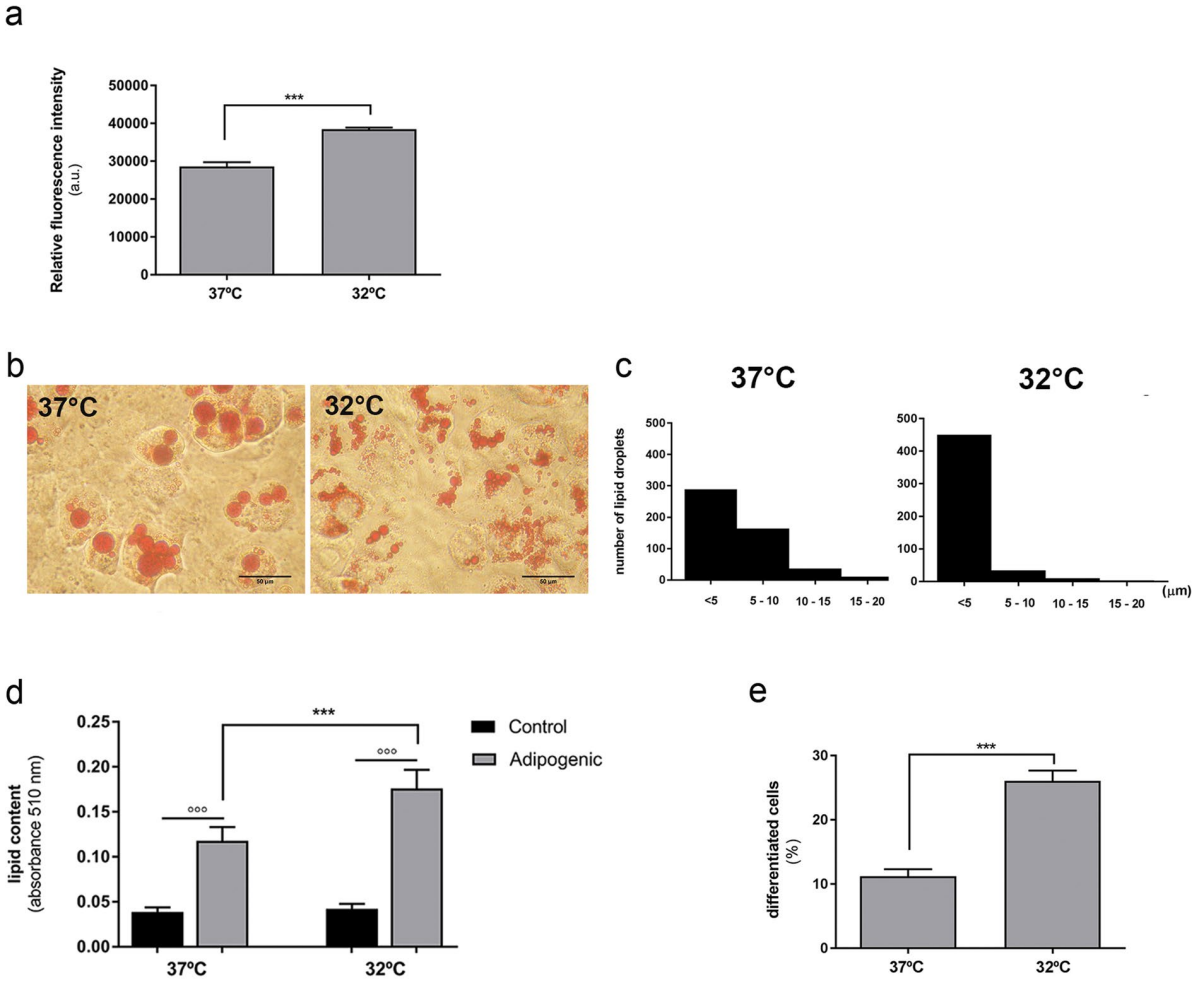
Cellular response of mMSCs exposed to adipogenic differentiation under standard or hypothermal conditions. (**a**) Metabolic activity measured in AD-treated cultures maintained at 32 and 37 °C. (**b**) ORO staining of cells after 9 days of differentiation at 32 and 37 °C, and (**c**) changes in LD size distribution (mean diameter). Scale bars, 20 μm. (**d**) Changes in lipid content per well in cells differentiated at 32 and 37 °C. ***Comparison of the same treatments at different temperature; °°°Comparison of different treatments at the same temperature. Statistical significance was set at p < 0.05. (**e**) Proportion of lipid-containing cells after differentiation at 32 °C and 37 °C. Measurements (**c** and **e**) were done on 50 randomly selected micrographs per condition (n = 3 individual experiments). Data are shown as mean ± SEM. Statistical significance was set at p < 0.05.

In order to investigate the influence of temperature on UCP1 expression in our model, cells were either maintained at 37 °C (standard temperature) or 32 °C (lower temperature) and subjected to adipogenic treatment. Using the Presto Blue assay, no deleterious effect was noticed in adipocytes differentiated at 32 °C and metabolic activity was increased (p < 0.001) (Fig. 2a). Cells were then subjected to ORO staining to further evaluate adipogenic differentiation. A large perinuclear lipid droplet (‘LD’) was observed in the cytoplasm of adipocytes differentiated at 37 °C, compared with numerous, smaller LDs located further away from the nucleus in adipocytes differentiated at 32 °C (Fig. 2b). Morphometric analysis of LD diameter confirmed the prevalence of larger LDs in adipocytes differentiated at 37 °C (Fig. 2c), which was accompanied by a lower total lipid content (Fig. 2d) and fewer differentiated cells (Fig. 2e). Taken together these results show increased metabolic activity, cellular differentiation and lipid content when incubated at a cooler temperature.

### Temperature-related changes in differentiated mMSC-derived adipocytes: increased UCP1 protein expression and leptin translocation to the nucleus

**Figure 3:**
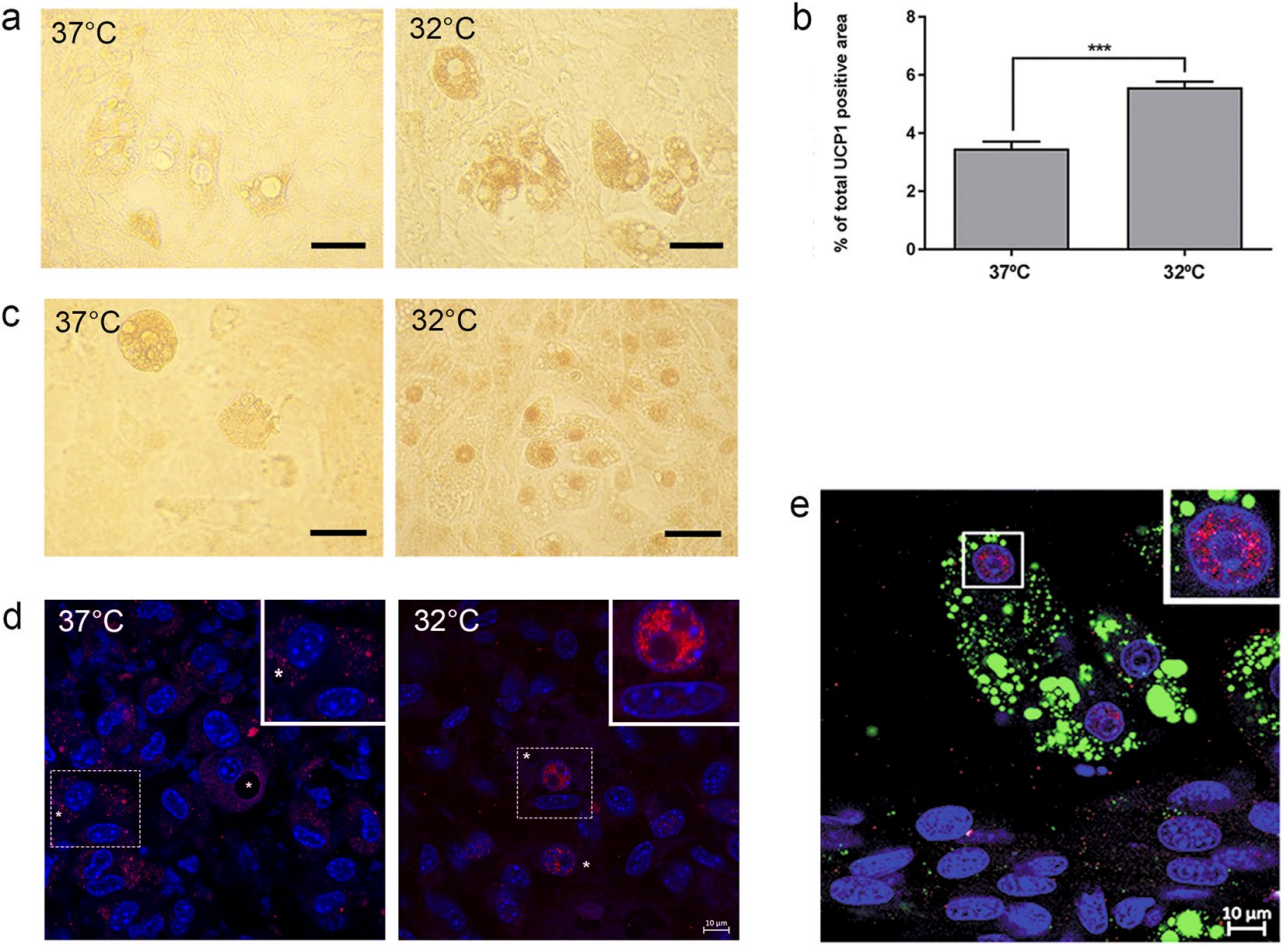
Temperature-related changes in differentiating mMSCs. (**a**) Detection of uncoupling protein (UCP)1 protein expression in adipocytes differentiated at 37 vs 32 °C visualized with 3, 3′-diaminobenzidine (DAB) as the chromogen. Scale bar: 20 μm. (**b**) Image quantification of UCP1-positive cell area. Data represent the mean ± SEM. Statistical significance was set at p < 0.05. (**c**) Leptin immunodetection in adipogenic cells in cultures differentiated in 37 vs 32 °C visualized with 3,3′-diaminobenzidine (DAB) as the chromogen. Scale bar: 20 μm. (**d**) Fluorescence image of leptin (red) cytoplasmic localization in adipocytes differentiated in 37 °C (left image) and leptin nuclear localization in adipocytes differentiated at 32 °C (right image). DAPI was used to identify nuclei (blue) and asterisks indicate LDs. Image inserts show enlarged view of adipocytes in boxed areas. Scale bar: 10 μm. (**e**) Representative fluorescence image of leptin nuclear localization (red) in adipocytes differentiated at 32 °C. DAPI was used to identify nuclei (blue) and BODIPY was used to identify LDs (green). Insert shows enlarged view of nucleus in boxed area. Scale bar: 10 μm. n = 3 individual experiments.

Next, the effect of temperature on UCP1 and leptin was analysed using single immunostaining (Fig. 3), and although UCP1 was observed in most differentiated adipocytes, exposure to a lower temperature strongly enhanced UCP1 abundance (Fig. 3a and b). Similarly, leptin was expressed in both adipogenic groups but surprisingly, unlike adipocytes differentiated at 37 °C where leptin was localized in the cytoplasm, in adipocytes differentiated at 32 °C leptin was found to be localized in the nucleus (Fig. 3c and d). This difference was confirmed by immunofluorescence detection using confocal microscopy imaging of serial sections with a thickness less than 1 μm (Fig. 3e) and making an orthogonal image through adipocyte nuclei (Supplementary Fig. S1). The same morphological changes related to LD size and their distribution were observed in primary mouse BM-derived adipocytes as well as in human adipose-derived stem cells (hADSCs) (Supplementary Fig. S2 and Supplementary Fig. S3). Furthermore, in both cell types UCP1 protein expression was increased (Supplementary Fig. S2) in adipocytes differentiated at 32 °C, and leptin was observed to be localized in the nucleus (Supplementary Fig. S3).

To determine whether this adipogenic phenotype acquired at 32 °C may be reversible as found *in vivo*^25^, cells differentiated at 32 °C were subsequently transferred to standard culture conditions (37 °C). These cells exhibited a morphology more similar to cells differentiated solely at 37 °C than that of cells differentiated at 32 °C (Supplementary Fig. S4a). Morphological changes were accompanied with changes in UCP1 and leptin expression. As can be seen from Supplementary Fig. S4b, cells transferred from 32 °C to 37 °C showed a pattern of UCP1 expression similar to that of cells differentiated at 32 °C (Supplementary Fig. S4c), as well as positive leptin reaction in nuclei, showing that the hypothermic cells retained their cold phenotype after rewarming (Supplementary Fig. S4d). Additionally, cells transferred from 37 to 32 °C showed the same pattern of UCP1 and thermotransient receptor potential ion channel subfamily V (TRPV)1 expression as cells differentiated at 32 °C (Supplementary Fig. S4e).

### Lower temperature conditions affect PGC-1α expression, the bioenergetic status and reduce coupling efficiency of MSC-derived adipocytes

**Figure 4:**
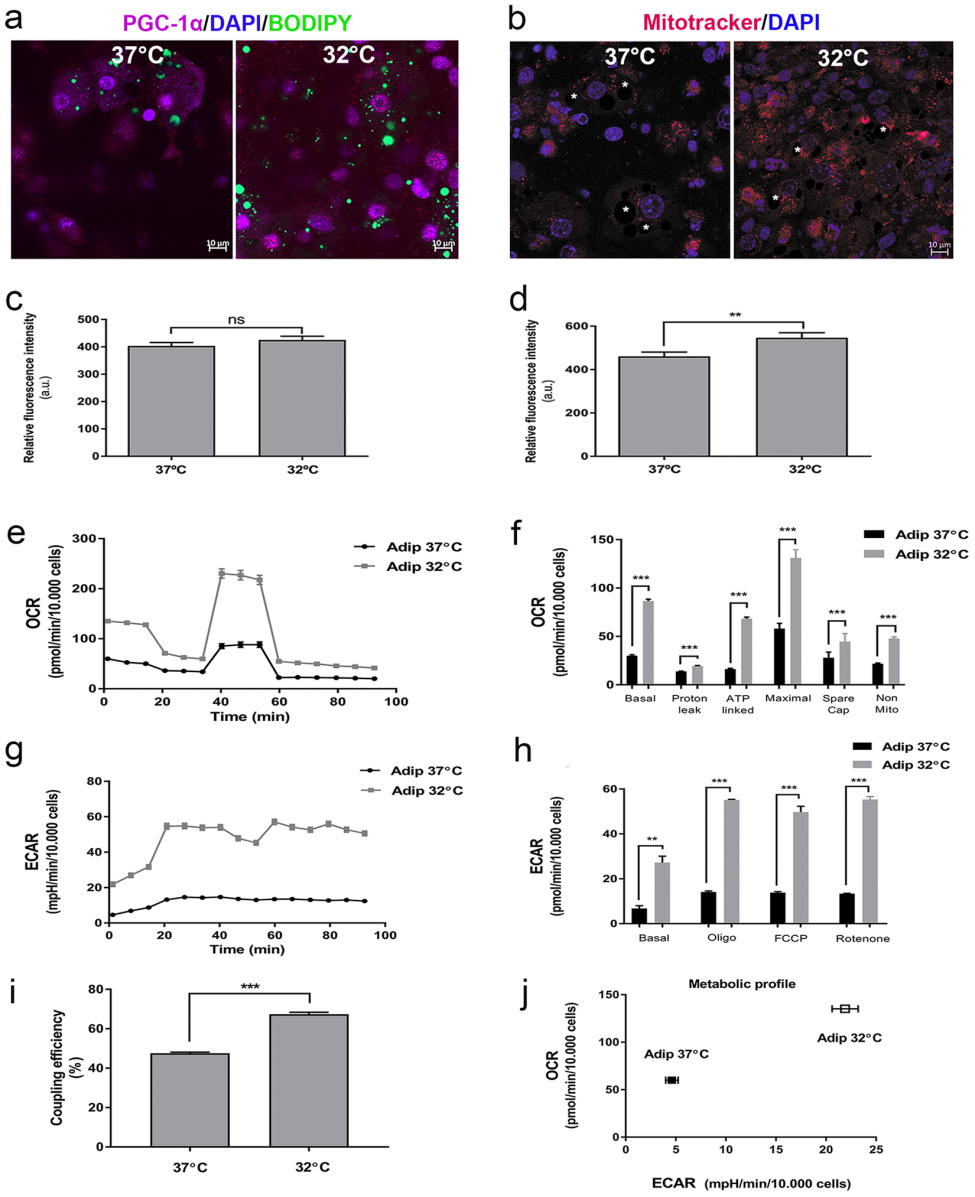
PGC-1α immunoexpression, mitochondrial localization and bioenergetics measurement during mMSCs differentiation under different temperature conditions. (**a**) Fluorescence image of PGC-1α (purple) in adipocytes differentiated at 37 °C and 32 °C. DAPI was used to identify nuclei (blue) and BODIPY was used to identify LDs (green). (**b**) mMSC-derived adipocytes stained with MitoTracker Orange CM-H_2_ TRos (red) and DAPI nuclear counterstain (blue). Asterisks – LDs. Scale bar: 10 μm. (**c**) Relative fluorescence intensity of MitoTracker staining was determined at randomly selected images, measuring relative fluorescence intensity on whole image (**d**) Relative fluorescence intensity of MitoTracker staining was determined at randomly selected ROI around LDs, at distance of 3 µm from LD’s membrane. Data represent the mean ± SEM. Statistical significance was set at p < 0.05. (**e**) OCR following differentiation of adipocytes at 37 and 32 °C, respectively. (**f**) Six parameters of mitochondrial function calculated from the bioenergetics profile (basal respiration, proton leak, ATP-linked respiration, maximal capacity, spare capacity and non-mitochondrial respiration). Data represent the mean ± SEM. Statistical significance was set at p < 0.05. (**g**) ECAR following differentiation of adipocytes at 37 and 32 °C, respectively. (**h**) Quantitative ECAR analysis of adipocytes differentiated at 37 and 32 °C, respectively following treatments with mitochondrial inhibitors. (**i**) Coupling efficiency indicating the proportion of respiratory activity used for ATP synthesis. Bars represent mean ± SEM. Statistical significance was set at p < 0.05. (**j**) The metabolic profile for both adipogenic groups was determined by plotting ECAR to OCR. Bars represent mean ± SEM. Statistical significance was set at p < 0.05. n = 6 individual experiments.

The dynamic process of adipocyte differentiation in both WAT and BAT is accompanied by changes in the mitochondrial number (mitochondrial biogenesis), shape and localization, and is under the control of several transcription factors including PGC-1α, a PPARγ coactivator and main regulator of mitochondrial biogenesis and oxidative phosphorylation^26,27^. PGC-1α immunostaining (Fig. 4a), and mitochondrial staining performed using MitoTracker Orange (Fig. 4b), revealed that cells differentiated at 32 °C exhibited strong nuclear PGC-1α signal compared to the cytoplasmic staining observed in cells differentiated at 37 °C. MitoTracker relative fluorescence intensity was slightly increased in adipocytes differentiated at 32 compared with 37 °C, although statistical significance was not reached (Fig. 4c). However, MitoTracker relative fluorescence intensity measured around LDs (Fig. 4d) was significantly enhanced in adipocytes differentiated at 32 °C, which confirmed that at lower temperature mitochondria were more frequently observed adjacent to the LDs compared with mitochondria in cells differentiated at 37 °C.

The bioenergetic status of cells was then investigated using a Seahorse XF96 flux analyser, and the overall respiratory responses (Fig. 4e) calculated for the six key regulators of mitochondrial function. Adipocytes differentiated at 32 °C had a markedly higher mitochondrial respiration under basal conditions and following oligomycin treatment (Fig. 4f), which results in chemically-induced uncoupling. Furthermore, ATP-linked oxygen consumption rate (OCR), maximal OCR, reserve capacity and non-mitochondrial OCR were all increased in adipocytes differentiated at 32 °C (Fig. 4f) and coupling efficiency (Fig. 4i), an indicator of the proportion of respiratory activity used for ATP synthesis, was reduced.

Simultaneously with the OCR detection, the extracellular acidification rate (ECAR) (Fig. 4g and h) was raised in cells differentiated at 32 °C. Following oligomycin treatment, when mitochondrial ATP synthesis is blocked, both adipocyte cultures were able to upregulate glycolysis to meet the demand for ATP. The ECAR response also increased more during carbonyl cyanide 4-(trifluoromethoxy) phenylhydrazone (FCCP) and rotenone/antimycin treatment in adipocytes differentiated at a low temperature (p < 0.001) (Fig. 4h). Plotting basal ECAR against OCR (Fig. 4j) summarised the effect of temperature which, when reduced, led to an increase not only in glycolysis, but also in oxidative phosphorylation, indicating more metabolically active cells.

### Mouse MSC-derived adipocytes exhibit characteristics of white, beige and brown adipocytes at protein and mRNA level

**Figure 5:**
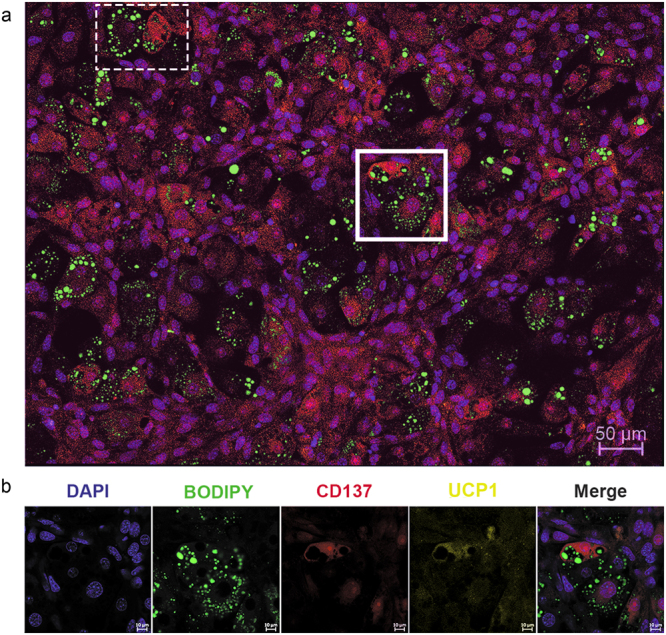
Expression of beige and brown markers in mMSCs-derived adipocytes differentiated at 32 °C. (**a**) Representative image showing the prevalence of beige cell-specific marker CD137 (red), multilocular phenotype and uncoupling protein (UCP)1 expression (yellow). DAPI was used to identify nuclei (blue) and BODIPY was used to identify LDs (green). The dashed square highlights difference in staining between two adjacent adipocytes. Scale bar: 50 μm. (**b**) Enlarged view of the square area, with individual and merged images showing adipocytes positive for both beige selective marker, CD137 (red) and brown cell-specific marker, UCP1 (yellow). Scale bars: 10 μm. n = 3 individual experiments.

**Figure 6:**
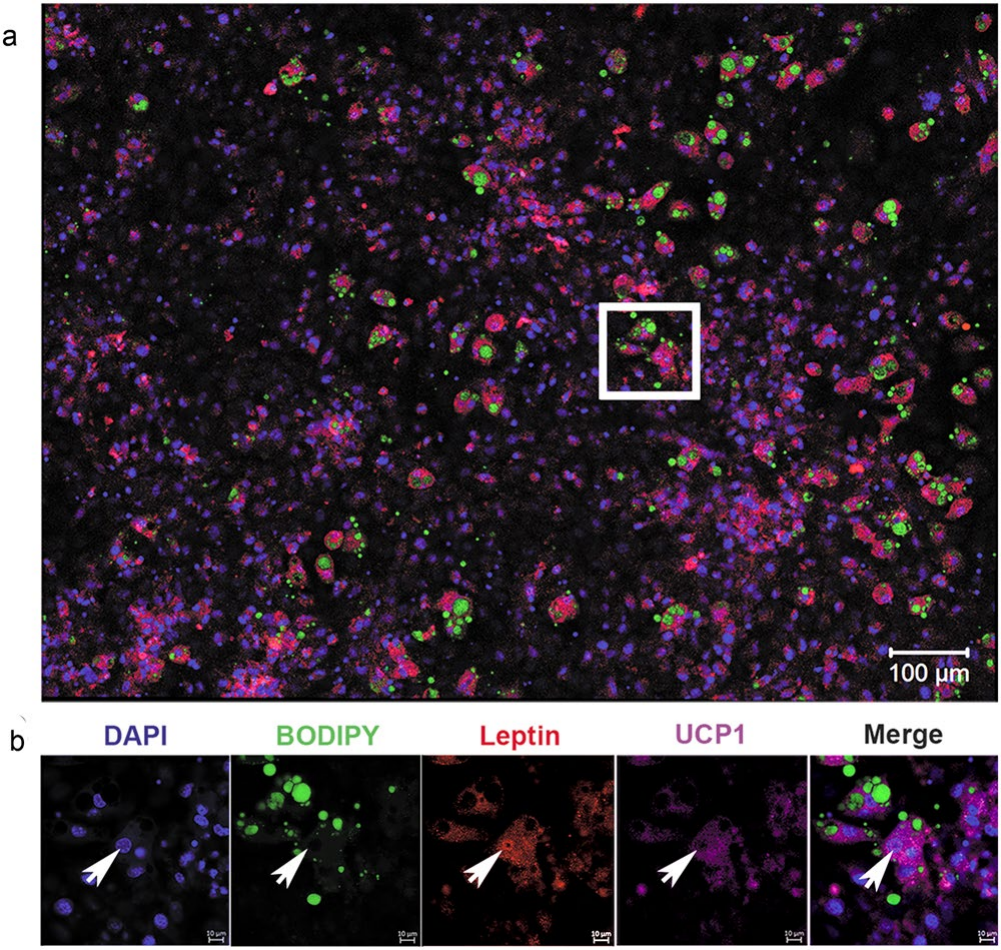
Leptin nuclear localization in UCP1^+^ mesenchymal stem cell-derived adipocytes differentiated at 32 °C. (**a**) Representative image showing double staining for leptin (red) and uncoupling protein (UCP)1 (purple). DAPI was used to identify nuclei (blue) and BODIPY was used to identify LDs (green). Scale bar: 100 μm. (**b**) Enlarged view of the square area, with individual and merged images showing adipocytes nuclei positive for leptin (red) and positive for UCP1 (purple). Arrows indicate nucleus positive for leptin and UCP1 cytoplasmic expression in the same cell differentiated at 32 °C. Scale bar: 10 μm. n = 3 individual experiments.

To determine whether our new model could generate adipocytes that exhibit the main features of beige cells, immunofluorescence detection was performed for CD137 and TMEM26 in undifferentiated mMSCs at 37 °C (Supplementary Fig. S5) and was found to be positive, with co-localisation on the cell membrane. Co-staining for CD137 and UCP1 showed that most of the adipocytes differentiated at 32 °C were positive for both (Fig. 5). Nuclear leptin localization in UCP1-expressing cells was further confirmed using double-immunofluorescence and confocal microscopy (Fig. 6). Additionally, this confirmed leptin and UCP1 co-expression in adipocytes differentiated at both 37 and 32 °C, with nuclear translocation of leptin only observed in the later.

**Figure 7:**
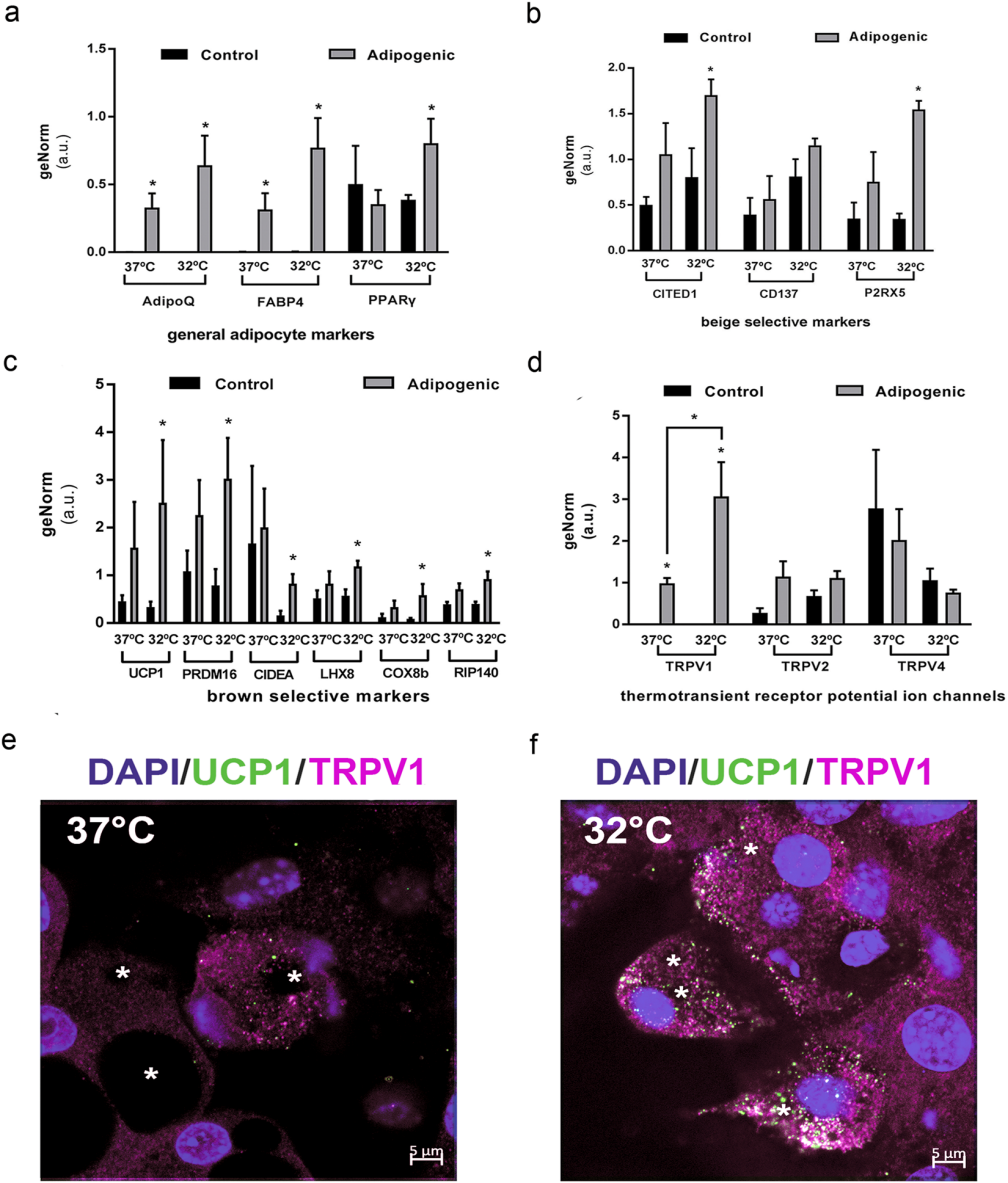
Real-time PCR analysis of gene expression during mMSCs differentiation under different temperature conditions. (**a**) Expression levels of general adipocytes markers: *AdipoQ*, *FABP4* and *PPARγ*. Data represent the mean ± SEM of three replicas. Statistical significance was set at p < 0.05. (**b**) Expression levels of beige lineage markers: *CITED1*, *CD137* and *P2RX5*. Data represent the mean ± SEM of three replicas. Statistical significance was set at p < 0.05. (**c**) Expression levels of brown lineage markers: *UCP1*, *PRDM16*, *CIDEA*, *LHX8*, *COX8b* and *RIP140*. Data represent the mean ± SEM of three replicas. Statistical significance was set at p < 0.05. (**d**) Expression levels of *TRPV1*, *TRPV2* and *TRPV4*. Data represent the mean ± SEM of three replicas. Statistical significance was set at p < 0.05. (**e** and **f**) Representative images showing double staining of TRPV1 (purple) and UCP1 (green) protein expression in adipocytes differentiated at 37 (**e**) and 32 °C (**f**) DAPI was used to identify nuclei (blue) and asterisks present LDs. Scale bars: 5 μm. n = 3 individual experiments.

Finally, gene expression was analysed with real-time PCR (Fig. 7) and, whilst *PPARγ* gene expression was detected in all samples, the expression of other general adipogenic markers, such as *AdipoQ* and *FABP4*, was found only in adipogenic-treated cultures. In addition, cells differentiated at 32 °C showed increased expression for each of these genes (Fig. 7a). Furthermore, leptin and leptin receptor expression was confirmed in all experimental groups (Supplementary Fig. S6). Similarly, beige-selective genes *CITED1*, *CD137* and *P2RX5* were expressed in all samples, and upregulated during differentiation at 32 °C (Fig. 7b). Notably, adipocyte differentiation at 32 °C promoted the upregulation of brown-selective genes, including *UCP1*, *PRDM16*, *LHX8*, *COXb8* and *RIP140*, but decreased the expression of *CIDEA* (Fig. 7c). Furthermore, expression of *TRPV*1, 2 and 4 was detected in BM-derived adipocytes differentiated at 37 and 32 °C. However, cells differentiated at 32 °C significantly increased the expression of *TRPV1*, supporting its involvement in mediating temperature-sensing mechanisms (Fig. 7d). Moreover, immunofluorescent staining confirmed TRPV1/UCP1 co-expression in adipocytes differentiated at 32 °C and 37 °C, with enhanced TRPV1 immunodetection in cells differentiated at 32 °C (Fig. 7e and f). Since TRPV1 is a Ca^2+^-permeable cation channel and its activation leads to calcium influx, cells were stained with Fluo-4, a Ca^2+^-sensitive dye. While signal for intracellular calcium was not detected in the majority of cells differentiated at 37 °C, most cells differentiated at 32 °C showed strong cytoplasmic Fluo-4 signal, consistent with TRPV1 activation in cells differentiated under low temperature conditions (Supplementary Fig. S7).

## Discussion

Here we present a novel mechanism to promote the browning of adipocytes with raised UCP1, which involves differentiating MSCs in pro-adipogenic medium at low temperature instead of standard 37 °C culture conditions. Browning was confirmed at the structural, protein and gene level, and critically, by the cells’ enhanced bionergetic capacity. We also observed a clear translocation of leptin to the cell nucleus of these multilocular cells expressing UCP1, a process that was enhanced at the lower temperature in both mouse and human cultures. These findings thus extend the observations from one previous report showing that 3T3-F442A adipocytes can directly sense temperature to activate thermogenesis, however the precise mechanism or relevance to stem cell differentiation has not been examined thus far. Leptin has previously been implicated in BAT function in the neonate^28^ where, in rodents, the post-partum maturation of the hypothalamic-pituitary axis is paralleled with raised leptin^29^, whilst in larger mammals’ exogenous leptin can have a thermogenic effect due to increased BAT function^30^. *In vivo*, leptin was found in both WAT and BAT, although leptin expression in BAT has been controversial^31–34^ and the dynamics of this process have not previously been explored. While the ability of BM-derived adipocytes to secrete leptin has been reported^23,35,36^, the dual UCP1/leptin expressing phenotype revealed here may indicate their functional diversity and potential to develop into both white and brown like cells. Given the recent emphasis on the precise function of leptin^37^, it may be this resides within the control of adipocyte fate during development rather than appetite control.

Cold exposure is one of the most important physiological stimulators of cellular energy metabolism, especially for BAT, and, *in vivo*, is known to increase proliferation, differentiation, UCP1 activity^1,38^ and the browning of white adipocytes^11^. Increased metabolic activity determined by Presto blue assay and Seahorse XF96 analysis was accompanied with raised adipogenesis, as determined using the ORO assay and changes in intracellular lipid concentration. Both the lipid content and the proportion of differentiated cells were raised under low temperature condition, followed by the appearance of numerous, small LDs, one of the most important morphological signs of browning^39,40^.

The increase in all markers of the browning process observed at the lower temperature is remarkable and includes a marked increase in all aspects of mitochondrial metabolism examined. This was accompanied with decreased LD size and increased lipid content compared to standard temperature conditions, indicating an enhanced rate of fat metabolism that could contribute to an enhanced release of leptin and its nuclear translocation. It has previously been shown that the conversion of thyroxine to triiodothyronine by the enzyme 5′monodeiodinase, which then binds to the nucleus, is essential in ensuring *UCP1* is transcribed in hypothermic rats^41,42^. The postnatal development of leptin within the brain^29,43^ also occurs at the same time as UCP1 increases within interscapular BAT^44^, thus suggesting that the regulation of nuclear function within the adipocyte could be a critical determinant of UCP1. The browning response induced with low temperature was associated with leptin translocation to the nucleus of UCP1 positive adipocytes, indicating a possible role for leptin in the browning process. The nuclear presence of leptin receptors has also been observed in a different cellular context^45^, suggesting a possible new regulatory role for leptin, a hypothesis consistent with its reported stimulatory effect on fatty acid oxidation^46^.

A key structural aspect of adipogenesis and the browning process is mitochondriogenesis^1^ when mitochondria are remodelled, changing shape and size, increasing in number and cristae formation^47^. However, we did not find a positive correlation between oxygen consumption and mitochondriogenesis but observed that LDs were smaller at a lower temperature, suggesting the enhancement of mitochondrial fatty acid oxidation, together with ATP utilisation. This is supported by the proximity of mitochondria and LDs observed in this study, suggesting a modified distribution to optimise substrate supply.

It is known that adipogenic differentiation promotes a transition from glycolytic metabolism to oxidative phosphorylation^48^, and an enhanced glycolytic rate has typically been interpreted as a consequence of reduced efficiency of ATP formation^49^. Here we found enhancement of two ATP-producing metabolic pathways, thus clearly indicating high energy demands of cells differentiated at 32 °C. Furthermore, the metabolic profile, UCP1 activation, increased lipid content and reduced coupling efficiency suggest raised fatty acid oxidation and lipogenesis. These two processes have already been reported to act in parallel. Simultaneous induction of specific genes involved in fatty acid oxidation and synthesis in brown, beige and white adipocytes occurs, enabling a continuous cycle of triglycerides hydrolysis and resynthesis^50^. These processes require large amounts of ATP and could, at least partly, be a further cause of increased metabolic activity as observed in our study.

The adipocytes differentiated at the cooler temperature also showed a high abundance of the beige cell marker CD137^10,14^, with half of the cells imaged showing positive staining. Uniquely, CD137 was detected in both control and adipogenic cells, and is indicative of the adipogenic potential of BM-derived MSCs. Furthermore, double-staining showed adipocyte differentiation at a low temperature promoted CD137/UCP1-positive cells. To provide an assessment of the transcriptional regulation that underlies all these changes, we performed gene expression analysis of known adipocyte markers. mMSC-derived adipocyte cultures expressed several white, brown and beige fat markers, with a prevalent molecular signature of beige adipocytes in both the non-differentiated and differentiated state. *P2RX5* is a beige adipogenic marker and its expression has been shown to increase in BAT and subcutaneous WAT in mice in response to chronic cold exposure^13^. On the other hand, *COX8b* has been described as a distinctive marker of WAT ‘browning’^15^. Upregulation of these genes, *COX8b*, *UCP1*, *PRDM16*, *P2RX5*, *CITED1* and *CD137* could suggest a transition from white to beige/brown adipocytes. Although known as a brown marker, *CIDEA* was downregulated here, which would be consistent with its direct inhibitory role on UCP1 activity^51^. There was only a limited effect of temperature on the level of gene expression for these markers, which suggests that the primary changes induced by cooling are occurring within the mitochondria and nucleus. It is also recognized that transcript abundance are not the main determinant of protein abundance^52^, and discrepancies were observed for UCP1 protein and gene expression^53^.

Increased TRPV1 expression showed that cold can exert its effect on adipocytes directly in cell-autonomous manner, activating a thermogenic program independent of the central nervous system. Interestingly, TRPV1 was recently identified as a novel regulator of leptin resistance^54^. Finally, MSC-derived adipocytes differentiated in standard culture conditions being exposed to low temperature exhibited an intermediate phenotype, with both characteristics of cells differentiated at 32 and at 37 °C, as they retained nuclear leptin and high UCP1 signal while holding larger LDs as an adaptive response. These intermediate characteristics indicate that MSC-differentiated cells exhibit phenotypic plasticity and suggest a possible cellular memory of previous environmental conditions. Further experiments could now be undertaken in order determine whether the process of transient thermal conditioning may improve the differentiation process, to analyse the role of TRPV1 in this response, and to determine whether TRPV1 inhibition reduces the phenotypic response induced by low temperature.

In summary, we show that BM-derived MSCs generate adipocytes that have a distinct phenotype with a prevalence of beige characteristics, which are able to undergo stimulated thermogenesis accompanied by leptin nuclear translocation. Same patterns of morphological changes, UCP1 expression and leptin translocation were observed in hADSCs and primary mouse BM-derived stem cells, indicating that MSCs provide a source of thermogenic beige adipocytes for human brown cell-based approaches to treat metabolic diseases.

## Methods

All laboratory procedures were carried out at The University of Nottingham under the United Kingdom code of laboratory practice (COSHH: SI NO 1657, 1988).

### Cell culture

Mouse mesenchymal cells (D1, ATCC CRL-12424) were grown and maintained routinely in standard medium containing low glucose Dulbecco’s modified Eagle’s medium (DMEM) supplemented with 10% fetal bovine serum (FBS), 1% penicillin/streptomycin, 1% L-glutamine and 1% non-essential amino acids. When cells reached 80% confluence, adipogenic differentiation was induced with adipogenic (‘AD’) medium prepared with standard medium supplemented with 1 μM dexamethasone (Cayman Chemicals, USA), 100 µM isobutylmethylxanthine (IBMX) (Sigma-Aldrich, UK), 1 μM rosiglitazone (Cayman Chemicals, USA), and 10 μg/ml insulin (Sigma-Aldrich, UK). All supplements were prepared according to the manufacturer’s guidelines. AD medium was used throughout the treatment period, as previously described^55,56^. Experimental cultures were treated for 9 days in two groups: cells differentiated under standard cell culture thermal conditions (37 °C), and cells differentiated at 32 °C^57–59^. Matching undifferentiated control cells maintained in standard medium were also grown at 37 and 32 °C. The same protocol was applied to human adipose-derived stem cells (hADSCs) (ThermoFisher UK, Cat. No R7788-115) and primary mouse BM-derived stem cells. For the analyses performed in this study, 3 batches of cells were analysed.

### Cell Viability Assay

Cell viability and metabolic activity was evaluated using the resazurin-based PrestoBlue Cell Viability kit, according to the manufacturer’s instructions. Cells were seeded in 24-well plates in three culture replicates and treated with standard and AD medium in appropriate temperature conditions. At the end of experiment, medium was replaced with a working solution of 10% v/v Presto Blue stock solution and incubated for 40 min. The fluorescent signals of 100 μl samples were measured at 560 nm excitation and 590 nm emission in triplicate using an Infinite M200 PRO plate reader and i-control software (Tecan, Switzerland). The measured fluorescent signal was expressed as arbitrary units (AU).

### Oil Red O staining

Cell monolayers were rinsed with phosphate buffered saline (PBS, pH 7.4), fixed with 4% paraformaldehyde, and analysed by ORO staining. After rinsing in PBS, cells were stained with 60% working ORO solution for 10 min, washed and subsequently counterstained with hematoxylin. Cells were imaged before incubation with 100% isopropanol to extract the incorporated stain. Absorption of extracted dye in triplicate was measured at 510 nm on an Infinite M200 PRO plate reader and i-control software (Tecan, Switzerland). The measured absorbance signal was expressed as AU. For all calculations and measurements, ImageJ software was used^60^.

### Morphometric and stereological analysis

The percentage of differentiated cells was estimated by dividing the total number of differentiated cells by the total number of all cells, using 50 randomly selected micrographs per condition (three biological replicates) at a final magnification of 400. The same images were used to evaluate Maximum Feret’s Diameter (MFD), the longest distance between any two points of the object. Ferret’s diameter is generally used in optical microscopy to measure the size of irregularly shaped particles^61^. In order to analyse LDs distribution, 500 LDs per group were measured. For all calculations and measurements, ImageJ software was used.

### Mitochondrial staining

Cells cultured for 9 days on coverslips were stained with MitoTracker Orange CM-H_2_TMRos (100 nM), according to the manufacturer’s protocol, and applied to cells in culture at 37 and 32 °C for 45 minutes before fixation. After PBS wash, coverslips were mounted on microscopic slides with Vectashield medium containing DAPI (VectorLabs, UK) and examined with a Zeiss fluorescence microscope. Mitochondria numbers were assessed using 20 randomly selected images per condition (three biological replicates). Relative fluorescence intensity was measured applying identical instrument settings. In addition, relative fluorescence intensity was measured around 50 LDs per group at 3 µm from the LDs.

### Calcium staining

Cells differentiated at 37 °C and 32 °C were incubated for 40 minutes with 2 µM Fluo-4 solution (Molecular Probes, F14201) as previously described^62^. Afterwards, cells were washed three times, fixed with 4% PFA and counterstained with DAPI. Images were taken with Nikon Eclipse 90i microscope.

### Immunocytochemistry

For immunostaining, cells seeded and differentiated on coverslips, were fixed with 4% ice-cold paraformaldehyde. Before immunostaining, cells were washed with PBS, permeabilised using 0.1% Triton X-100 (Sigma Aldrich, UK) in PBS and incubated with 5% normal goat serum or fetal bovine serum in PBS (depending on secondary antibody host) for 60 min at room temperature. Samples were then incubated in the appropriate primary antibody for 90 min and washed in PBT (PBS + 0.1% Tween20, Sigma Aldrich) before incubation with the appropriate fluorophore-conjugated secondary antibodies for 60 min. After extensive washing in PBT, samples were mounted with Vectashield medium containing DAPI (VectorLabs) and examined with Zeiss Elyra PS.1 microscope. If biotinylated secondary antibodies (VectorLabs) were used, signal was visualized with 3,3′-diaminobenzidine (DAB) as the chromogen (VectorLabs), samples were mounted on slides using DPX and examined with a Nikon microscope. All antibodies used in this study are presented in Table 1 (see Supplementary information).

### Densitometric analysis of UCP1 positive area

Twelve fields per condition (three biological replicates) were randomly selected at x400 magnification and the UCP1 positive areas were calculated using the image processing software ImageJ. The images were converted to HSB stack with saturation settings for MinThreshold and MaxThreshold to segment UCP1 positive areas. The percentage of area within the threshold range was measured.

### Super-resolution imaging in structured illumination mode

For super-resolution imaging, cells mounted with CitiFluor (CF3) medium (Agarscientific, UK) were imaged using a Zeiss Elyra PS.1 microscope, in structured illumination mode (SIM), with the following settings: objective Plan-Apochromat 63 x /1.4 Oil DIC M27, filter set LBF −488/561, cmos camera exposure time 20 ms. Two imaging tracks were set up in fast frame mode, which alternates the excitation lasers (solid state 488 nm and 561 nm, at 20 and 10% laser power settings, respectively). In the fast frame mode, images from the two channels were acquired almost simultaneously with an exposure time difference of 20 ms between the red and green channel. Additionally, channel alignment was performed by scanning 100 nm beads with the same settings to ensure the precise localization. Image processing and channel alignment was done using the manufacturer’s software (https://www.zeiss.com/microscopy/int/products/microscope-software/zen-lite.html).

### Gene expression analysis

Total RNA from cells, WAT, BAT and liver tissue samples was extracted, normalized to 0.5 µg/µl and reverse transcribed to cDNA as previously described^63^. Quantitative PCR was performed using either SYBR Green Taq polymerase master mix or TaqMan universal master mix with murine-specific oligonucleotide primers (Eurofins) or FAM-MGB TaqMan probes against a cDNA gene standard curve to verify the efficiency of the reaction (≥95%) and with appropriate negative controls using the Step One Plus Q-PCR system and v.2.2 software (Applied Biosciences). Gene expression was determined using GeNorm normalization algorithm against two selected reference genes (stability value M = 0.47), *TBP* (TATA sequence binding protein) and acidic ribosomal protein subunit P0 (*RPLP0*) using GeNorm software version 3.5 (Primer Design Ltd). *UCP1*, *PRDM16*, *COX8b*, *P2RX5*, *TRPV1*, *TRPV2* and *TRPV4* gene expression level was determined using a TaqMan probe (BioRad TaqMan Gene Expression assays; assay qHsaCEP0050537, Mm00712556_m1, qMmuCIP0034367 and qRnoCIP0024301, qMmuCIP0031313, qMmuCIP0035343 and qMmuCIP0032629, respectively). Primers used in this study are listed in Table 2 (see Supplementary information).

### Seahorse Assay

Cells were seeded into the XFe96 Microplates (Seahorse Bioscience) to measure the OCR, an indicator of mitochondrial respiration, and the ECAR, an indicator of glycolysis. Prior to the start of the Seahorse XF Cell Mito Stress Test Assay, cells differentiated at either 37 or 32 °C were washed 3 times and incubated in XF-Basal Medium supplemented with 10 mM glucose, 1 mM sodium pyruvate, and 2mM L-glutamine. OCR and ECAR readings were taken over time under basal conditions and after the addition of mitochondrial inhibitors (2.5 μM oligomycin, 0.8 μM FCCP and 1 μM rotenone/antimycin) at 37 and 32 °C, respectively. OCR was determined using the Seahorse Wave 2.4 XF-96 software and the data obtained for each condition were normalized to the cell number per well and expressed as the OCR in pmol/min/10^4^ cells. OCR and ECAR were determined using the Seahorse Wave 2.4 XF-96 software and the data obtained for each condition were normalized to the cell number per well and expressed as the OCR in pmol/min/10^4^ cells. Measures of mitochondrial respiration (basal respiration, ATP-linked respiration, proton leak, maximal and reserve capacity) were derived by the sequential addition of oligomycin, FCCP and rotenone/antimycin. First, the basal respiration rate was calculated by subtracting the residual OCR determined after the addition of rotenone/antimycin. ATP-linked respiration was determined from the difference between basal OCR and OCR following oligomycin addition. The difference in OCR between rotenone/antimycin and oligomycin represented the amount of oxygen consumed due to proton leak respiration. Maximal OCR was determined by subtracting the OCR after rotenone/antimycin addition from the OCR induced by FCCP. Last, the mitochondrial reserve capacity was calculated by subtracting basal respiration from maximal reserve capacity.

ECAR was measured simultaneously with OCR using the same conditions as described previously^49^ and glycolytic rate was determined by monitoring extracellular pH induced by the change in lactate. Similarly to OCR, basal ECAR refers to the ECAR measured before injection of rotenone/antimycin. To determine the influence of low temperature on oxidative phosphorylation and glycolysis, basal OCR was plotted against basal ECAR. Lastly, coupling efficiency, an indicator of the proportion of respiratory activity used to make ATP, was determined by calculating the percentage of OCR immediately following the oligomycin treatment with the final baseline value.

### Data and software availability

All data were analysed by Student’s t test. For data that was not normally distributed the nonparametric Mann Whitney test was used to evaluate the statistical significance between two treatment groups. Statistical significance was accepted at p < 0.05, with *p < 0.05; **p < 0.01; ***p < 0.001. Errors bars plotted on graphs are presented as the mean ± SEM. Data were analysed using Graph Pad Prism Software (https://www.graphpad.com).

## Electronic supplementary material

Supplementary Figures
