# Supplementary material for: Low temperature exposure induces browning of bone marrow stem cell derived adipocytes in vitro
Source: Sci Rep. 2018 Mar 21;8:4974. doi: 10.1038/s41598-018-23267-9 (PMC5862957; doi:10.1038/s41598-018-23267-9)
Supplement: Supplementary file 1 — Supplementary Figures [file 41598_2018_23267_MOESM1_ESM.docx]

**Supplementary information:**

**Low temperature exposure induces browning of bone marrow stem cell derived adipocytes in vitro**

Ksenija Velickovic^1^, Hilda Anaid Lugo-Leija^1^, Ian Bloor^2^, James Law^2^, Harold Sacks^4^, Michael Symonds^2,3*^ and Virginie Sottile^1*^

^1^Wolfson Centre for Stem Cells, Tissue Engineering and Modelling (STEM), School of Medicine, University Park - CBS Building; University of Nottingham; Nottingham, NG7 2UH; United Kingdom.

^2^The Early Life Research Unit, Division of Child Health, Obstetrics and Gynaecology, and ^3^Nottingham Digestive Disease Centre and Biomedical Research Centre School of Medicine, University Hospital; University of Nottingham; Nottingham, NG7 2UH; United Kingdom.

^4^VA Endocrinology and Diabetes Division, VA Greater Los Angeles Healthcare System, and Department of Medicine, David Geffen School of Medicine; University of California; Los Angeles, CA, 90073; USA.

Correspondence to: virginie.sottile@nottingham.ac.uk, michael.symonds@nottingham.ac.uk

**Tables:**

**Supplementary Table 1.**

| **Table 1.** List of antibodies used in this study. | | | |
| --- | --- | --- | --- |
| **Primary antibody** | **Host** | **Source** | **Dilution** |
| Anti-UCP1 | Rabbit | Abcam (ab10983) | 1:500 |
| Anti-UCP1 | Goat | Novus Biologicals (NB100-2828ss) | 1:100 |
| Anti-Ob | Rabbit | Santa Cruz (sc-843) | 1:100 |
| Anti-CD137 | Rabbit | Abcam (ab203391) | 1:100 |
| Anti-TMEM26 | Rabbit | Novus Biologicals (NBP2-27334ss) | 1:100 |
| Anti-PGC-1α | Rabbit | Abcam (ab54481) | 1:100 |
| Anti-CD137 | Rabbit | Abcam (ab203391) | 1:100 |
| Anti-TRPV1 | Rabbit | ThermoFisher Scientific (PA1-29421) | 1:100 |
| **Secondary antibody** | **Host** | **Source** | **Dilution** |
| Anti-rabbit IgG Alexa 488 | Goat | ThermoFisher Scientific (A11008) | 1:1000 |
| Anti-goat IgG Alexa 647 | Goat | ThermoFisher Scientific (A21244) | 1:500 |
| Anti-goat IgG Alexa 647 | Donkey | ThermoFisher Scientific (A21447) | 1:500 |
| Anti-goat IgG Alexa 488 | Donkey | Abcam (ab150129) | 1:1000 |
| Anti-rabbit IgG Alexa 594 | Goat | ThermoFisher Scientific (R37117) | 1:500 |
| Anti-rabbit IgG Alexa 633 | Goat | ThermoFisher Scientific (A21070) | 1:500 |
| HRP Anti-Rabbit IgG | Goat | Vector Laboratories (PI-100) | 1:300 |

**Supplementary Table 2.**

| **Table 2.** List of primers used in this study. | | |
| --- | --- | --- |
| **Target gene** | **Forward Primer (5´-> 3´)** | **Reverse Primer (5´-> 3´)** |
| Adiponectin | AACTTGTGCAGGTTGGATGGC | TTCTCTCCCTTCTCTCCAGGA |
| CD137 | CGTGCAGAACTCCTGTGATAAC | GTCCACCTATGCTGGAGAAGG |
| CIDEA | TGACATTCATGGGATTGCAGAC | GGCCAGTTGTGATGACTAAGAC |
| CITED1 | AACCTTGGAGTGAAGGATCGC | GTAGGAGAGCCTATTGGAGATGT |
| Leptin  Leptin Receptor  (short receptor isoforms 1-3) | CCAGGATGACACCAAAACC  AGCTAGGTGTAAACTGGGACA | TGGACAAACTCAGGAGAGG  GCAGAGGCGAATCATCTATGAC |
| FABP 4 | TGGAAGCTTGTCTCCAGTGA | AATCCCCATTTACGCTGATG |
| LHX8 | AAGAGGCAGCTGTCCACG | GAAGCTGGTCCGAGCTCTTT |
| PPARγ | GCCGAGTCTGTGGGGATAAA | GTGAGACATCCCCACAGCAA |
| RIP140 | TCCCCGACACGAAAAAGAAAG | ACATCCATTCAAAAGCCCAGG |
| RPLP0 (Ref Gene) | CTGGAGAAACTGCTGCCTCA | AGTGTTCTGAGCTGGCACA |
| TBP (Ref Gene) | CCTTGTACCCTTCACCAATGAC | ACAGCCAAGATTCACGGTAGA |


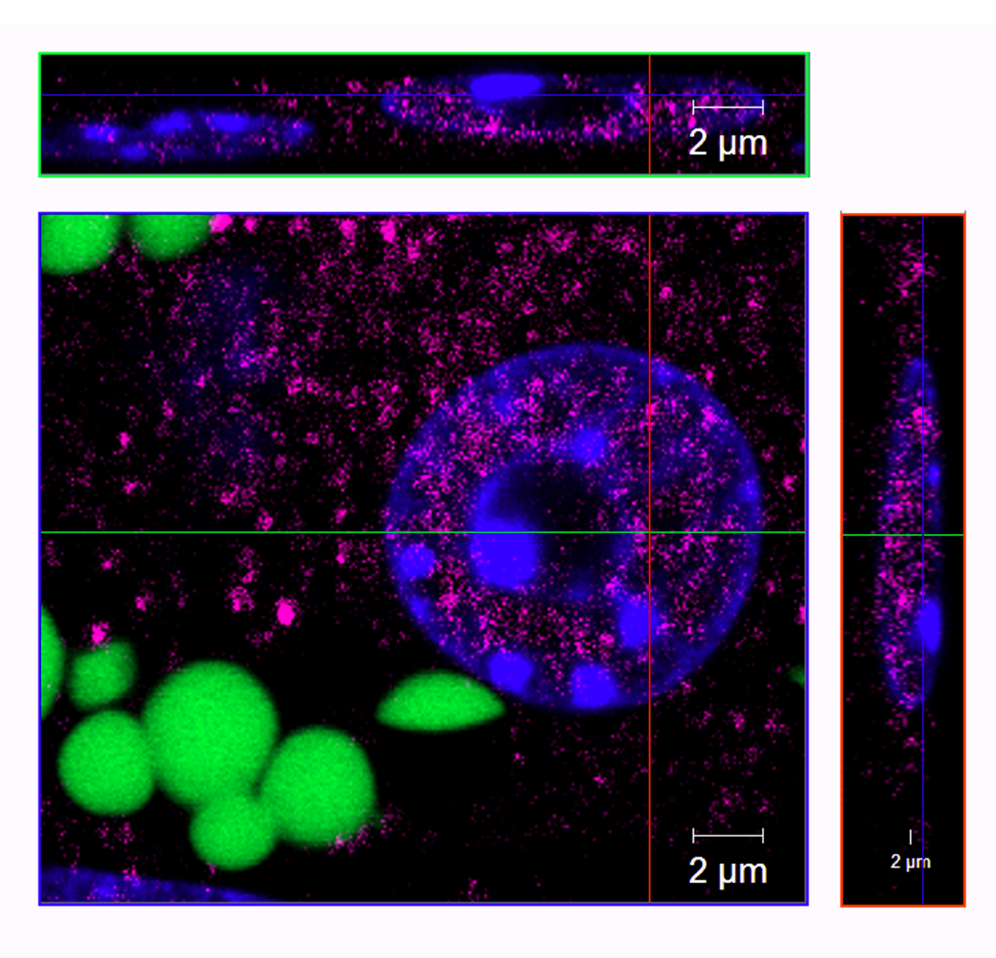


**Supplementary Fig. S1.** Image from a confocal z-stack with orthogonal views of leptin (purple) localization in adipocytes differentiated at 32⁰C. DAPI was used to identify nuclei (blue) and BODIPY was used to identify LDs (green). Scale bar: 2 μm.

**
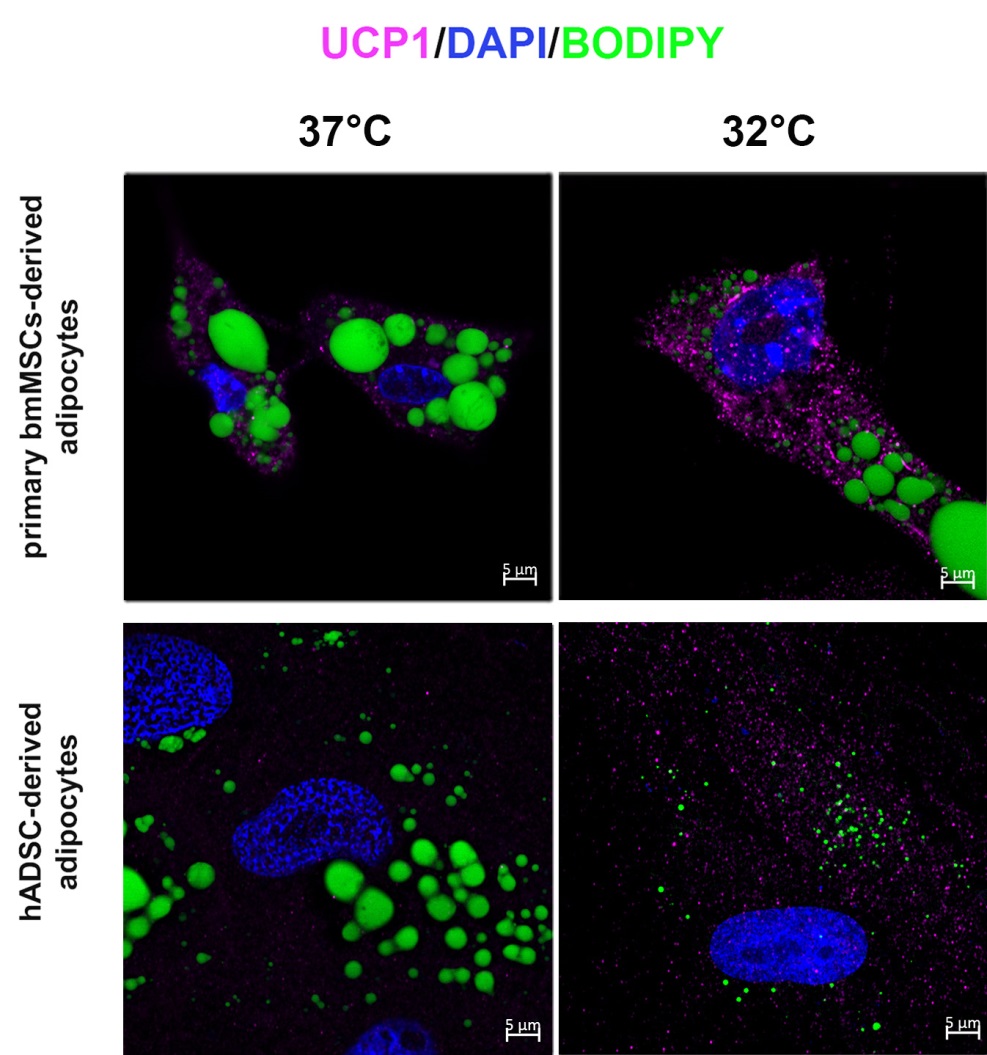
**

**Supplementary Fig. S2.** Temperature-related changes in differentiating mouse primary bone marrow (BM) mesenchymal stem cells (MSC) derived and human adipose-derived cells (hADSCs). Detection of *UCP1* (purple) protein expression in adipocytes differentiated at 37 vs 32°C. DAPI was used to identify nuclei (blue) and BODIPY was used to identify LDs (green). Scale bar: 5 μm. n = 3 individual experiments.

**
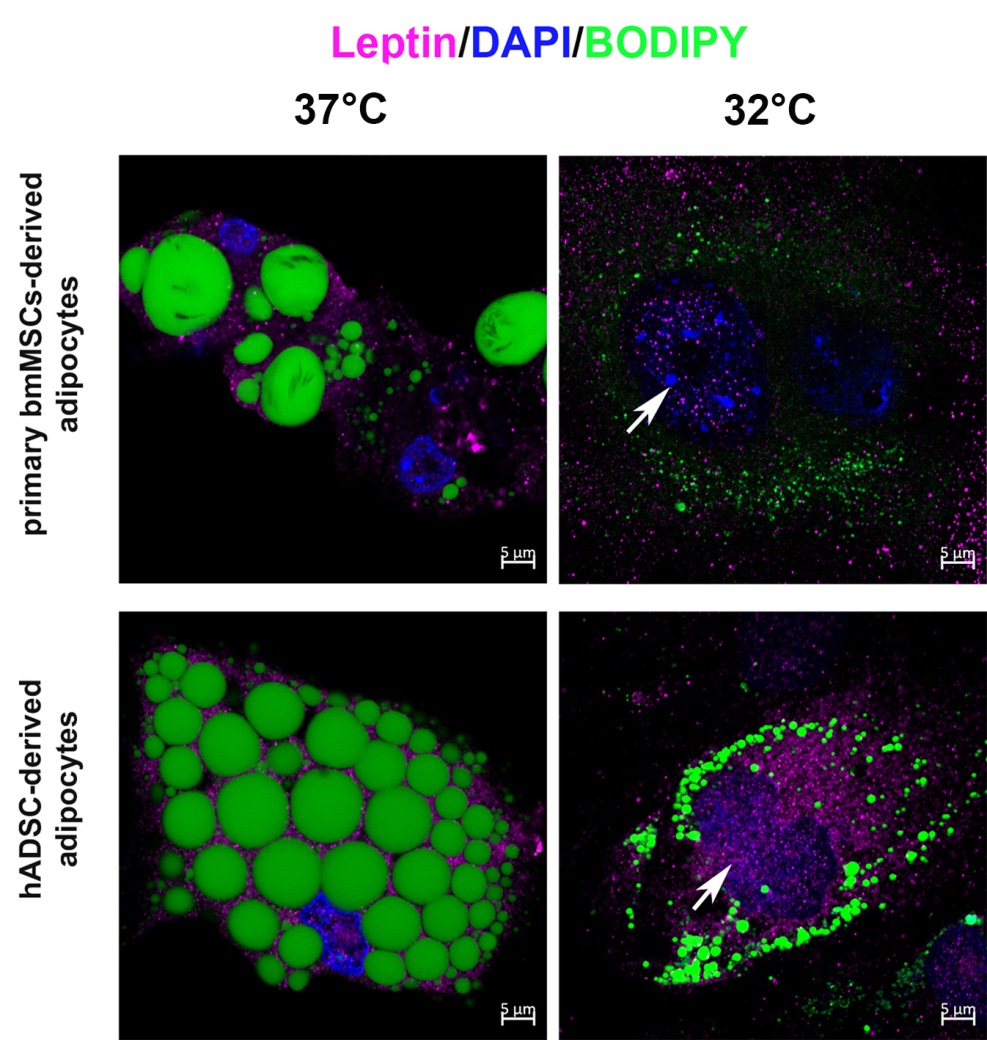
**

**Supplementary Fig. S3.** Temperature-related changes in differentiating mouse primary bone marrow (BM) mesenchymal stem cells (MSC) derived and human adipose-derived cells (hADSCs). Detection of leptin (purple) protein expression in adipocytes differentiated at 37 vs 32°C. DAPI was used to identify nuclei (blue) and BODIPY was used to identify LDs (green). Arrows indicate leptin nuclear localization in cells differentiated at 32°C. Scale bar: 5 μm. n = 3 individual experiments.

**
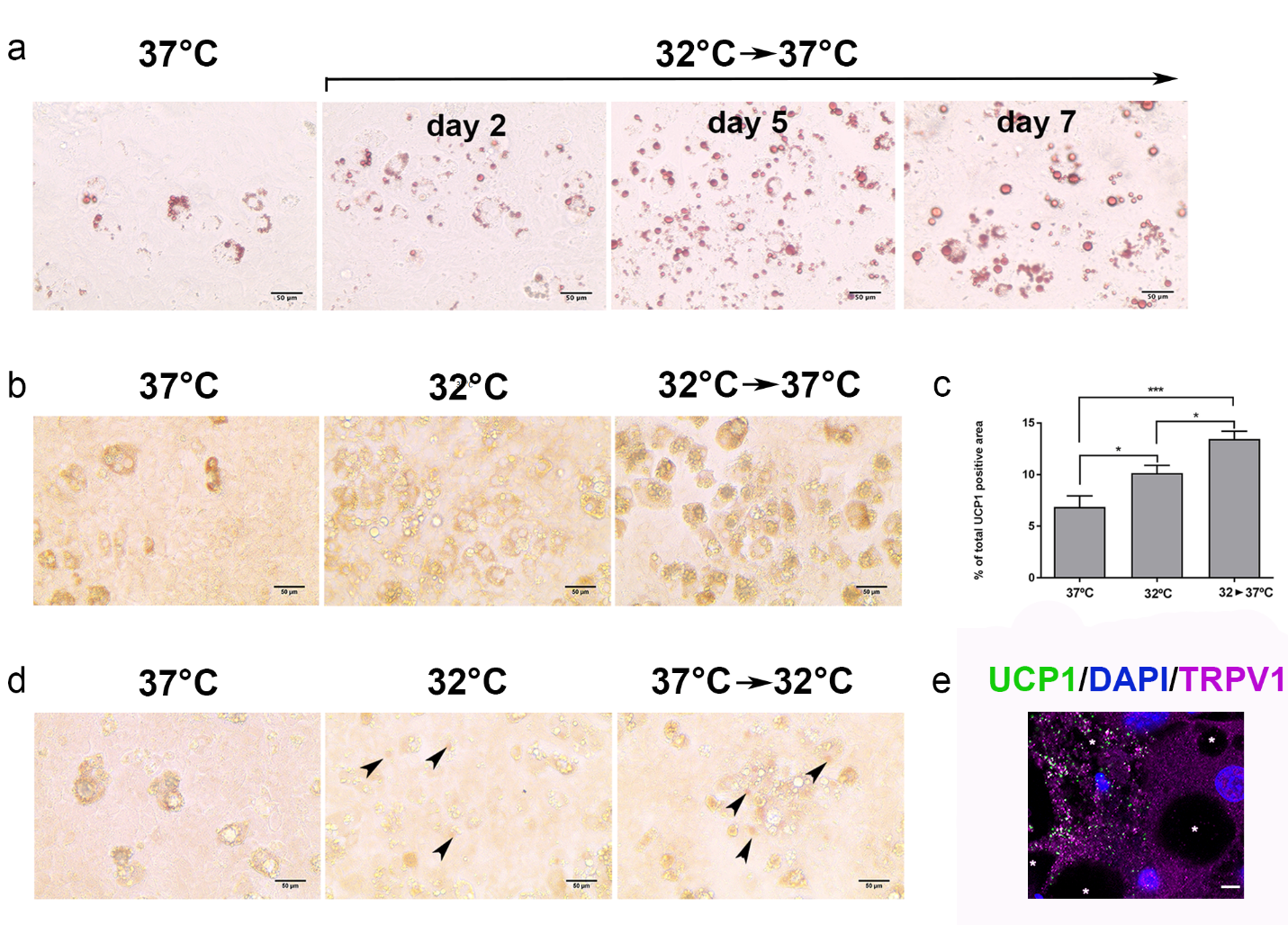
**

**Supplementary Fig. S4.** Morphological changes in cells transferred from 32 to 37°C and UCP1 expression. **(a)** mMSCs were differentiated at 32°C for seven days, transferred to 37°C for the next seven days and stained with ORO. During this period, cells achieved intermediate phenotype with large LDs and enhanced differentiation. **(b)** Morphological changes are accompanied by variation in *UCP1* protein expression. Higher level of *UCP1* expression could be seen in groups differentiated at 32°C and transferred to 37°C. **(c)** Image quantification of *UCP1*-positive cell area. Data represent the mean ± SEM. Statistical significance was set at p<0.05. **(d)** Leptin expression in adipocyte nuclei in groups differentiated at 32°C and transferred to 37°C. Scale bars: 50 μm. **(e)** Representative image showing double staining of *TRPV1* (purple) and *UCP1* (green) protein expression in adipocytes transferred from 32 to 37°C. DAPI was used to identify nuclei (blue) and asterisks indicate LDs. Scale bar: 5 μm. Images representative of 3 individual experiments.

**
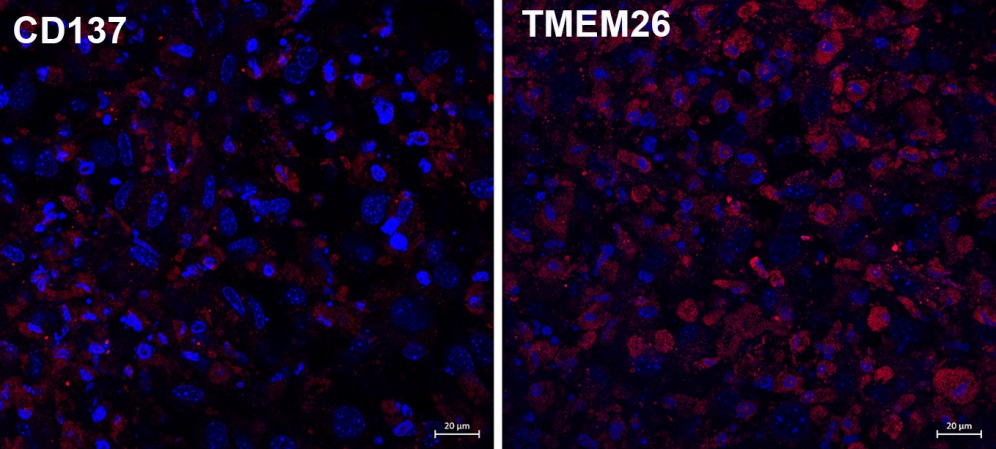
**

**Supplementary Fig. S5.** CD137 and TMEM26 immunodetection in undifferentiated mMSCs at 37°C. Fluorescence image of beige selective markers showing cell membrane expression. DAPI was used to identify nuclei (blue). Scale bars: 20 μm. Images representative of 3 individual experiments.


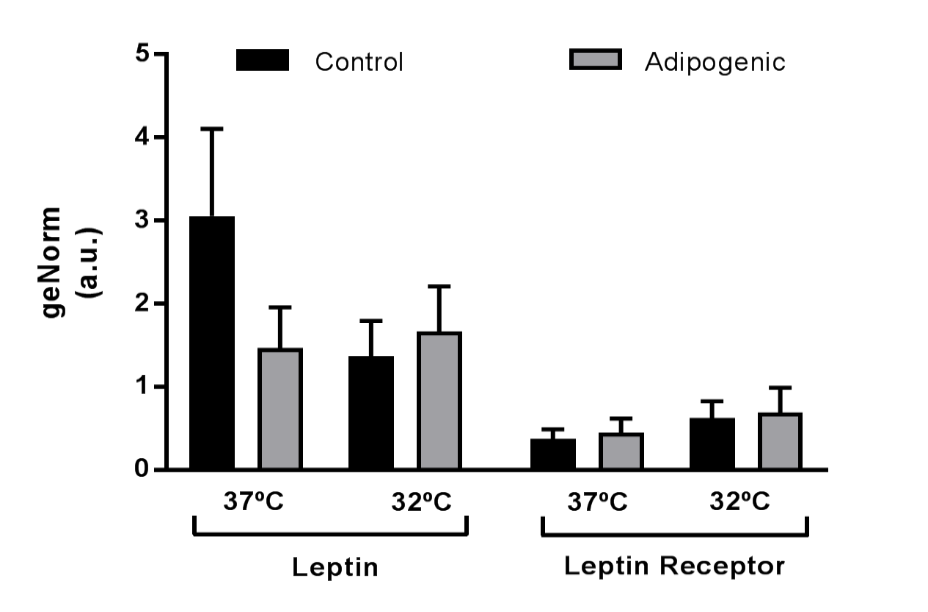


**Supplementary Fig. S6.** Real-time PCR analysis of Leptin and Leptin Receptor gene expression during mMSCs differentiation under different temperature conditions at day 9. Data represent the mean ± SEM of three replicates. Statistical significance was set at p<0.05.


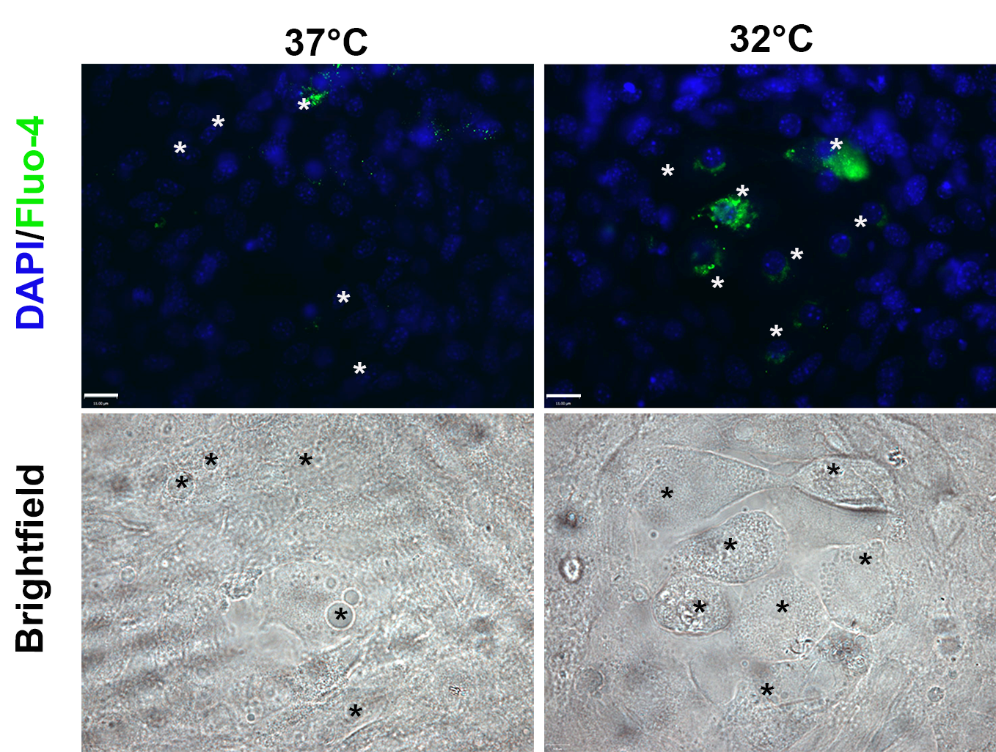


**Supplementary Fig. S7.** Temperature-related changes in differentiated mouse MSCs stained with Fluo-4. Detection of Ca^2+^ (green) in adipocytes differentiated at 37 vs 32°C after 9 days. DAPI was used to identify nuclei (blue). Stars indicate LDs-laden adipocytes. Scale bar: 10 μm.
